# Is There a Limitation of RECIST Criteria in Prediction of Pathological Response, in Head and Neck Cancers, to Postinduction Chemotherapy?

**DOI:** 10.1155/2013/259154

**Published:** 2013-09-11

**Authors:** V. Patil, V. Noronha, A. Joshi, Vamshi Muddu Krishna, S. Juvekar, Gauri Pantvaidya, Pankaj Chaturvedi, D. Chaukar, Supreeta Arya, Aswari Patil, B. Bhosale, A. Dongre, A. K. Dcruz, K. Prabhash

**Affiliations:** ^1^Department of Medical Oncology, Tata Memorial Hospital, Room 1108, HBB, E Borges Road, Parel, Mumbai, Maharashtra 400012, India; ^2^Department of Radiodiagnosis, TMH, Mumbai, India; ^3^Department of Head and Neck Surgical Oncology, TMH, Mumbai, India; ^4^Department of Pathology, TMH, Mumbai, India

## Abstract

This study studied the coorelation between radiological response to induction chemotherapy and acheivement of pCR or near pCR. It was a retrospective analysis in which all patients who received NACT from 2008 till april 2012 were subjected to inclusion criteria. Coorelation analysis was performed between CR + PR and acheivement of pCR or near pCR. Twenty four patients were identified.The primary site of tumor was oral cavity in 19 patients (79.2%), maxilla in 2 patients (4.2%), laryngopharynx in 2 patients (4.2%) and oropharynx in 1 patient (4.2%). The clinical stage was stage IVA in 16 patients ( 66.7%) and IVB in 8 patients (33.3%). The overall response rates ie a combination of CR and PR was seen in 11patients (45.8%). The pCR was seen in 15 patients (62.5%) and rest had near pCR. There was no linear coorelation between radiological size decrement and tumor response. On coorelation analysis the spearman correlation coefficent was −0.039 (*P* = 0.857). This suggest that presently used radiological response criterias for response assesment in head and neck cancers severly limit our ability to identify patients who would have pCR or near pCR.

## 1. Introduction

 RECIST (response evaluation criteria in solid tumors) criteria have been widely used for response assessment both in general clinical practice and clinical trials [1]. The criteria have been designed to be objective and reproducible. However, in RECIST, the focus is on unidimensional imaging and volumetric changes are not included. This lacuna was accepted in the 2009 update of RECIST (version 1.1); however, unidimensional imaging remained the standard as volumetric assessment was not considered to be standardised appropriately, and the measurements were often time-consuming and required special techniques [2]. Furthermore, RECIST criteria are easier to apply in well-defined lesions, as in metastatic nodules [3, 4]. The application of these criteria in complex, irregularly shaped tumors like head and neck cancers might be more difficult. This assumes significance in situations where response to initial therapy would dictate the next treatment protocol, especially in patients being treated with curative intent [5–7]. Prasad et al. have shown that the treatment response was graded differently based on volumetric measurement as opposed to unidimensional imaging of tumor burden in head and neck cancers [8].

Thus, reliance on RECIST criteria alone for assessing response might have an implication on the treatment algorithm in head and neck cancers. Induction chemotherapy is one of the treatment options in patients with head and neck cancers [9–12]. The aim of induction chemotherapy is to decrease tumor load and to eradicate potential systemic micrometastases. The ideal response would be a pathological complete response (pCR) indicating chemosensitive disease. Logically, patients achieving pCR should have better overall outcomes. This was confirmed by Licitra et al.; those patients who achieved pCR post-induction therapy had a 5-year survival of 78% as opposed to 40% in those who did not achieve pCR [13].

At our centre, patients with borderline resectable tumors as determined by radiological imaging in a multidisciplinary clinic are treated with neoadjuvant chemotherapy (NACT). Based on the radiological and clinical responses, further treatment could be definitive surgery or concurrent chemoradiotherapy. In our initial experience of oral cavity patients, there was a perceived discordance between response rates assessed by axial imaging and clinical resectability achieved at the end of induction. 28.5% of patients with radiological partial response (PR) were deemed clinically unresectable while, conversely, 35% of patients with stable disease (SD) later underwent curative resection (sent for publication).

Considering the discordance between the radiological response rates and surgical decisions and the proven significance of pathological response, we decided to analyse the correlation between radiological decrement in size of primary and pathological decrement in size of primary tumor. The aim was to study the positive predictive value (PPV) and negative predictive value (NPV) of radiological response to induction chemotherapy (complete response (CR) + partial response (PR)) as a predictor for achievement of pCR. We gave importance to NPV as if a patient would be denied a potentially curative treatment on the basis of its result; then we feel that there should be a high NPV of such a test.

## 2. Materials and Methods

We performed a retrospective analysis of our neoadjuvant chemotherapy (NACT) database which included all patients sequentially attending the head and neck services Medical Oncology Outpatient Department with the data being entered prospectively. All patients who received NACT from August 2008 till April 2012 were screened. Those patients who underwent axial imaging (either computed tomography or magnetic resonance imaging) not more than 1 month prior to starting NACT and reassessment with the same imaging modality within 1 month of completion of NACT were selected. Among this cohort, the patients who underwent surgery within 6 weeks of last radiological assessment and had achieved either a pathological complete response or near pathological complete response at site of primary were selected for the present analysis.

The pre- and post-NACT axial images for all patients were reviewed by the radiologist, and the response was assessed according to RECIST criteria. The symptomatic response was documented in all cases and defined as a percentage reduction in size of the tumor as perceived by the patient at the end of NACT. For the present analysis, analogous to the RECIST, a perceived decline of more than 30% but less than 100% was termed as PRp, 100% decline was termed as CRp, a reduction not more than 30% or a progression in size no more than 20% was termed as SDp, and a progression in size of more than 20% was termed as PDp. Pathological complete response was defined as absence of foci of tumor cells at the local site (pCR). Near pathological complete (pPRL) response was defined as either presence of scanty residual foci of cells or reduction of tumor size below 1 cm pathologically.

A total of 1200 patients had received NACT in head and neck cancers at our center within the last 4 years. The whole population of 1200 patients were subjected to the selection criteria. Unfortunately pathological CR rates are quite low in very locally advanced oral cancers (which constituted 90% of our whole population); hence, only twenty-four patients were eligible for the present analysis. The median age was 48 years (27–69 years). There were 19 males and 5 females. Twenty-two patients (91.7%) had visible and palpable external swelling which was perceived by the patients. All patients had ECOG performance score of one. The primary site of tumor was oral cavity in 20 patients (83.4%), maxilla in 2 patients (8.4%), and laryngopharynx in 2 patients (8.4%). In oral cavity, the most common primary was in the buccal mucosa in 16 patients (66.7%). All patients had squamous cell carcinoma. The T stage distribution was T1 in 1 patient (4.2%), T3 in 5 patients (20.9%), T4a in 14 patients (58.3%), and T4b in 4 patients (16.7%). The N stage distribution was N0 in 8 patients (33.3%), N1 in 5 patients (20.9%), N2 in 7 patients (29.2%), and N3 in 4 patients (16.7%). The clinical stage was stage IVA in 16 patients (66.7%) and IVB in 8 patients (33.3%). All of these patients were discussed in a multidisciplinary clinic, and a decision to administer NACT in view of borderline resectability was made.

SPSS version 16 has been used for statistical analysis. Descriptive analyses have been performed. A 2 × 2 cross-tabulation was done as shown in Table 2. Due to poor performance of the traditional test of concordance we moved ahead to study whether there existed a relationship between decrement in size of the tumor radiologically and a decrement in size of the tumor pathologically. A scatter plot was plotted to study the relationship between percentage decrement in radiological size and percentage decrement in pathological size. Since all body tissues are essentially three-dimensional, we tested whether reduction not only in single dimension (linear fit) but also in volume (cubic fit ) or area (quadratic fit) of the tumor could correlate more consistently with the response. Then a correlation analysis was performed between these parameters. For correlation analysis, patients with pCR and those with scanty residual foci of cells were taken as having 100% decrement. In those patients with decrement below 1 cm of tumor, the percentage of pathological decrement was calculated according to the formula given below.

1. Pathological decrement in size of tumor = maximum size in axial slice in axial imaging done prechemotherapy − maximum size pathologically in post chemo resected specimen.
2. Proportional pathological decrement = pathological decrement in size of tumor/maximum size in axial slice in axial imaging done prechemotherapy.
3. Percentage pathological decrement = proportional pathological decrement × 100.

Scatter plot was plotted separately to study the relationship between percentage decrement in size reported by patient and percentage decrement in pathological size. Then a correlation analysis was performed between the two parameters. The correlation coefficients are being reported with respect to the effect size as given by Cohen [14]. Spearman correlation was done as the sample size was small and skewed so assumptions and conditions for use of Pearson product moment correlation were violated.

## 3. Results

The chemotherapy schedule received was a 3-drug combination of docetaxel, cisplatin, and 5 FU in 10 patients (41.7%); and 2-drug combination of taxane and platinum in 14 patients (58.3%). The median numbers of cycles received were 2 (range 2-3).

**Figure 1:**
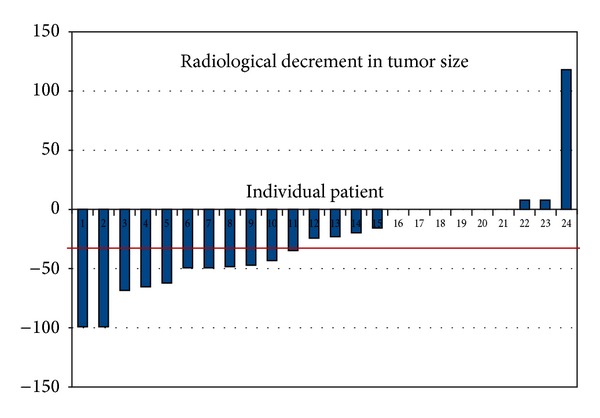
Radiological decline in tumor swelling in % according to RECIST criteria (primary tumor and not nodal). On *x*-axis are the individual patients and on *y*-axis is the tumor decline in percentage. Please note that if the tumor has decreased in size it has been charted along the negative values on *y*-axis.

**Figure 2:**
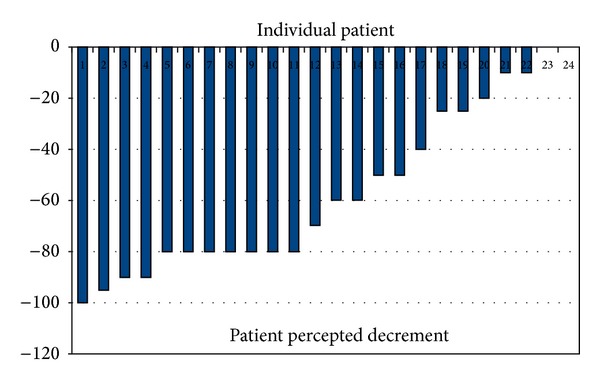
Patient percepted decline in tumor swelling (primary tumor and not nodal). On *x*-axis are the individual patients and on *y*-axis is the tumor decline in percentage. Please note that if the tumor has decreased in size it has been charted along the negative values on *y*-axis.

The waterfallplot of decrement in local tumor size according to radiological assessment and according to patient's assessment of decrement in size of the swelling is shown in Figures 1 and 2, respectively. The radiological response rates are shown in Table 1. The overall response rate which included CR and PR was 45.8% (11 patients). The patient's perception of decline in swelling was documented in 22 patients, as only these patients had an externally visible and palpable swelling. CRp was seen in 1 patient (4.5%), PRp was seen in 16 patients (72.7%), and SDp was seen in 5 patients (22.8%). Postoperatively, pCR was seen in 15 patients (62.5%).

The cross-tab between pCR and CR + PR combination has been shown in Table 2. It can be observed in the table that a significant percentage of patients (8/13) with radiological SD + PD eventually turned out to have pCR. The positive predictive value, negative predictive value, sensitivity, and specificity of radiological response (combination of CR + PR) to predict for pCR were 45%, 38%, 38%, and 45%, respectively.

**Figure 3:**
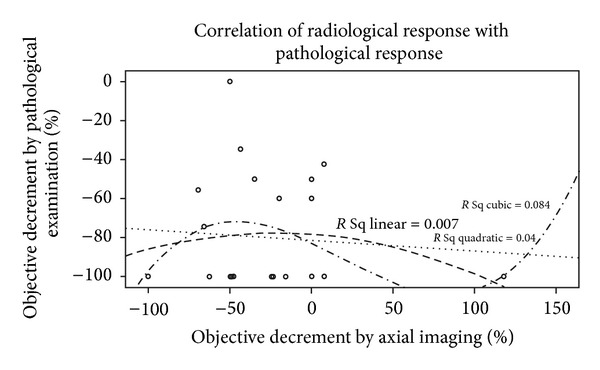
The scatter plot of the relation between % decrement in pathological specimen and % radiological decrement in size of primary.

**Figure 4:**
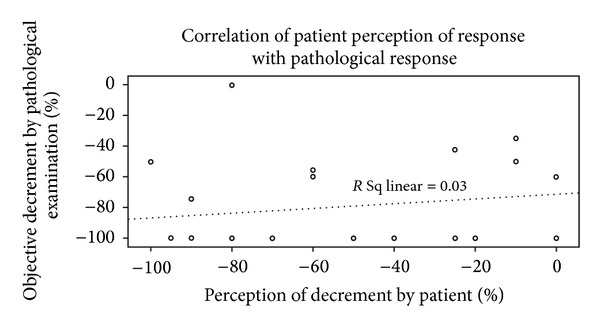
The scatter plot of the relation between % decrement in pathological specimen and % decrement in size of primary according to patients perception.

The scatter plot of the relation between percentage decrement in pathological specimen and percentage radiological decrement in size is shown in Figure 3. On correlation analysis the Spearman correlation coefficient was −0.039 (*P* = 0.857). There was no linear, quadratic, or cubic relation found between these parameters. The scatter plot of the relation between percentage decrement in pathological specimen and percentage decrement in patient reported size is shown in Figure 4. On correlation analysis the Spearman correlation coefficient was 0.174  (*P* = 0.415). Though the correlation coefficient was numerically better and was unidirectional for patient reported decline in size, it was not statistically significant.

## 4. Discussion

**Figure 5:**
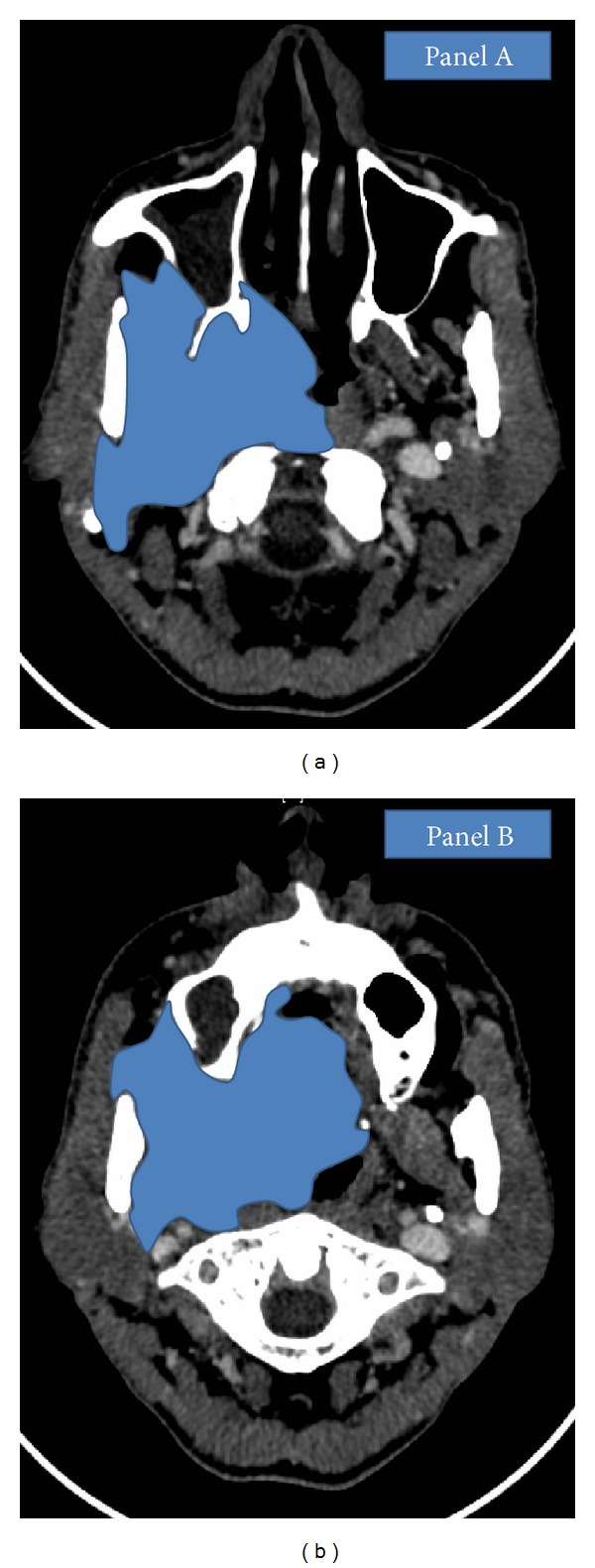
Complex shape of locally advanced head and neck cancers as depicted in (a) and (b).

The intent of RECIST is to provide objective, uniform, reproducible, and standard guidelines which could be applied in a uniform manner while reporting chemotherapy response rates [1, 2]. RECIST criteria are now more frequently used than WHO criteria as these are easier to apply (single dimension measurement), lesions have been defined as measurable and nonmeasurable, the numbers of target lesions have been defined, the type of imaging has been defined, and a specific cut-off for progression has been defined [4, 15]. In RECIST the unidimensional measurement in the preintervention and postintervention scans is not necessarily the same. The longest dimension in the axial image has to be used. This implies that the longest dimension selected in the postintervention scan need not be the same as used in the preintervention scan [1, 2, 15]. Head and neck cancers arise in areas of complex anatomy with complex fascial planes, bones, and soft tissue. Figures 5(a) and 5(b) depict one example of complex shapes often seen in head and neck cancers.

In the present paper, we have shown that there is no correlation between the radiological response and the postoperative pathological response. Furthermore, both the positive and negative predictive values of radiological response (combination of CR + PR) for prediction of pCR are below 50%. This implies that radiological response alone cannot accurately judge the actual pathological response within the tumor. Consequently, relying on radiological criteria alone could conceivably limit the ability to distinguish responders from nonresponders. These results are in accordance with our previous experience where nearly one-third of patients with partial response did not undergo surgery while nearly one-third of patients with stable diseases according to radiological criteria underwent curative surgery.

**Figure 6:**
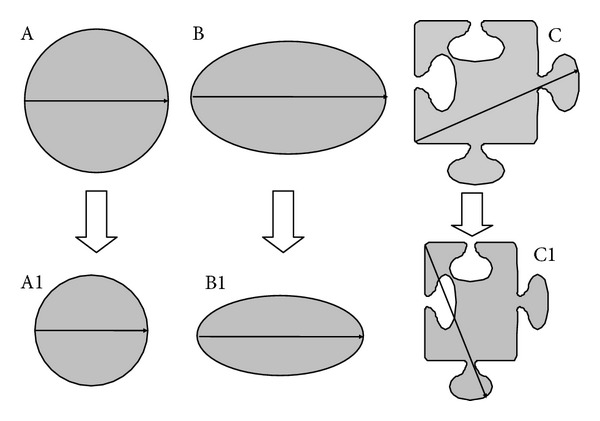
Scenario A: in a simple shape-like sphere or cylinder, the axial image would seem like a circle. In such tumors when there is a centripetal shrinkage due to the simple geometry application of change in unidimensional imaging would reflect the change in area on the axial slice and also the change in volume when the whole three-dimensional structure is taken into account. Scenario B: again in this situation the shape is simple; however, the tumor underwent a centrifugal shrinkage mainly in one dimension. The change in area on the axial slice or the change in volume (when the whole structure is accounted for) is not proportionately depicted to be change in unidimensional dimension in accordance with RECIST. However, a change is still depicted. Scenario C: in complex irregular shapes, as seen in the images, due to use of longest dimension for comparison in prechemotherapy and postchemotherapy scan even though, there has been a decrement in the area of the tumor on axial slice (which would in turn reflect a decrement in volume); this change has not been picked up by presently used radiological response criteria. As seen in Figure 5 head and neck cancers do not uncommonly have such shapes.

A hypothetical situation illustrates the limitations of RECIST in head and neck cancers. In scenario A, Figure 6, the target lesion is circular in axial image and shrinks centripetally following chemotherapy. Thus, unidimensional measurement would proportionally predict for the change in volume. Similarly in scenario B, a simple shaped tumor undergoes centrifugal shrinkage. The change in unidimensional measurement would predict for change in volume in this case too. Scenario C depicts a complex irregularly shaped target lesion. Posttherapy, there is an obvious reduction in the volume of the lesion appreciated visually. However, due to irregular shape, unidimensional measurement alone, in accordance with RECIST, will fail to show a satisfactory response [16]. Such scenarios are not uncommon in locally advanced head and neck cancers where NACT has been used and could be the reason for discrepancy between radiological and pathological size decrement.

Discordance between pathological and morphological remissions has been seen in other solid malignancies, notably germ cell tumors. Fibrous tissue, treatment-related necrosis, and nonviable tumors may not get resorbed completely [17, 18]. Change of RECIST criteria to functional imaging based criteria like PERCIST (positron emission tomography response criteria in solid tumors) might overcome this fallacy [19]. Targeted therapy represents another situation where lack of morpholic response does not imply lack of efficacy [20]. In our analysis, we included patients with pCR as it appears to be a surrogate marker for improved survival. The aim was to correlate the pCR with the radiological remission. However, it was felt by the investigators that many patients would have nonmeasurable disease so we decided to include patients with disease on gross pathological examination below 1 cm. Expanding the selection to include patients with residual disease on gross pathological examination below 1 cm enhanced our ability to see the correlation between decrement in size suggested by axial imaging versus decrement observed on pathology.

All the scans of the patients included in this study were initially reported by dedicated oncoradiologists and reviewed by oncoradiologists of the head and neck multidisciplinary clinic. Pathological response was similarly assessed by a pair of pathologists. There are limitations in the present study. The number of patients is small. Though majority of the scans (75%) were done in our institute, the remaining were done at other centres. Since all the scans were later reviewed by our radiologists and found to be of adequate quality, we believe that this may not have significant effect on the results. At present, we do not have long-term follow-up data for these patients. In spite of these shortcomings, we believe our findings highlight the fallacies intrinsic to using RECIST criteria alone for the evaluation of response to chemotherapy in head and neck cancers. A combined assessment tool which includes physical examination, visual inspection (with pre- and postphotographs), surgical opinion, patients' perceived change in the tumor, and radiological responses is needed to accurately identify patients who derive with induction therapy and can have operable disease posttreatment. A comparable paradigm could be the use of separate criteria as have been developed for gastrointestinal stromal tumor (GIST) and hepatocellular carcinoma [20–23].

## 5. Conclusions

pCR is an important surrogate marker for long-term survival in patients with head and neck cancers. Presently used radiological response criteria for response assessment appear to be inadequate in identifying true pathological response. This suggests an urgent need for developing new criteria for predicting response in head and neck cancer patients receiving neoadjuvant chemotherapy.

## Figures and Tables

**Table 1:** Radiological response.

	Frequency	Percentage
CR	2	8.30%
PR	9	37.50%
SD	12	50.00%
PD	1	4.20%

**Table 2:** Cross tabulation between pathological response and radiological response.

	pCR	pPR	Total
CR + PR	5	6	11
SD + PD	8	5	13

Total	13	11	Grand total = 24
